# Ecotoxicological Effects of Environmentally Relevant Concentrations of Nickel Nanoparticles on Aquatic Organisms from Three Trophic Levels: Insights from Oxidative Stress Biomarkers

**DOI:** 10.3390/jox15040112

**Published:** 2025-07-04

**Authors:** Alberto Teodorico Correia, Eduardo Motta, David Daniel, Bruno Nunes, José Neves

**Affiliations:** 1 Instituto de Ciências Biomédicas Abel Salazar (ICBAS), Universidade do Porto (UP), Rua Jorge Viterbo 228, 4050-313 Porto, Portugal; 2 Centro Interdisciplinar de Investigação Marinha e Ambiental (CIIMAR/CIMAR), Terminal de Cruzeiros do Porto de Leixões, Avenida General Norton de Matos S/N, 4450-208 Matosinhos, Portugal; 3 Faculdade de Ciência e Tecnologia (FCT), Universidade Fernando Pessoa (UFP), Praça 9 de Abril 349, 4249-004 Porto, Portugal; 4 Centro de Estudos do Ambiente e do Mar (CESAM), Universidade de Aveiro, Campus de Santiago, 3810-193 Aveiro, Portugal; 5 Departamento de Biologia, Universidade de Aveiro (UA), Campus de Santiago, 3810-193 Aveiro, Portugal; 6 Faculdade de Ciências da Saúde (FCS), Universidade Fernando Pessoa (UFP), Rua Carlos da Maia 296, 4200-150 Porto, Portugal

**Keywords:** aquatic ecotoxicology, nickel nanoparticles, oxidative stress biomarkers, lipid peroxidation, species- and tissue-specific responses

## Abstract

This study investigated the ecotoxicological impacts of environmentally relevant concentrations (0.05, 0.50, and 5.00 mg/L) of nickel nanoparticles (Ni-NPs) by assessing oxidative stress biomarkers. The worm *Hediste diversicolor*, the bivalve *Mytilus* spp., and the fish *Sparus aurata* were chronically exposed to Ni-NPs for 28 days, and glutathione S-transferases (GST), catalase (CAT), and thiobarbituric acid reactive substances (TBARS) levels were measured to evaluate biochemical responses. GST activity increased in *H. diversicolor* and the liver of *S. aurata*, suggesting a key role for this enzyme in Ni-NPs detoxification. CAT activity was inhibited in the digestive gland of *Mytilus* spp. at the highest Ni-NPs concentration, indicating possible disruption of antioxidant defense. TBARS levels rose significantly in the gills of *Mytilus* spp. exposed to high Ni-NP concentrations, suggesting oxidative damage beyond detoxification capacity. In contrast, TBARS decreased in the digestive gland of *Mytilus* and in *H. diversicolor*, possibly due to compensatory upstream antioxidant responses. These findings indicate that each species exhibits distinct adaptive responses to Ni-NP exposure. Overall, this study highlights the need to consider species- and tissue-specific responses when performing ecotoxicological risk assessments of nanomaterials.

## 1. Introduction

Aquatic ecosystems cover more than two-thirds of our planet and play a crucial role in stabilizing the global climate, in addition to providing a wide range of ecosystem services [1]. However, human activities have led, over recent decades, to a steady increase in the pollution of these ecosystems, causing detrimental impacts [2,3,4]. At present, aquatic ecosystems are increasingly exposed to numerous pollutants and their toxic effects, leading to the degradation of water quality and posing risks to aquatic life [5]. The extensive array of pollutants arising from anthropogenic activities includes, among others, pesticides, pharmaceutical compounds, personal care products, plastics, detergents, hormones and nanoparticles [6,7,8]. Nanoparticles (NPs), particularly those of metallic nature, are particles smaller than 100 nm exhibiting unique properties that distinguish them from their larger counterparts [9]. These distinct physicochemical characteristics have enabled their application across a wide array of sectors, including electronics, pharmaceuticals, medicine, food, cosmetics, packaging, waste management, and wastewater treatment—key components of modern life [9]. The widespread and continually increasing use of metallic NPs across diverse sectors has underscored the urgent need to understand the consequences of their release into various environmental compartments, particularly regarding potential risks to human and ecosystem health [10,11,12]. This need has been further emphasized by recent findings from ecotoxicological studies on specific metallic NPs, including ZnO-NPs [13,14], Ag-NPs [15,16], CeO_2_-NPs [17,18], TiO_2_-NPs [19,20], and Cu-NPs [21,22], among others.

Nickel nanoparticles (Ni-NPs) are metallic nanomaterials of significant industrial interest due to their capacity to catalyze the reversible hydration of CO_2_ into carbonic acid under environmental conditions [23]. Ni-NPs have experienced notable growth in both production and application. Industry estimates suggest annual production volumes of metallic NPs ranging from 200 to 500 metric tons, with Ni-NPs comprising a significant fraction—particularly in catalytic and magnetic applications [24]. The global market for Ni-NPs is expected to reach USD 759.7 million by the end of 2030 [25]. As with nanoparticles in general, Ni-NPs can enter ecosystems through various pathways, including direct emissions, leaching from soil, dry or wet atmospheric deposition, or other routes [26]. Given their critical role in carbon capture technologies and mineralization processes [27], it is anticipated that Ni-NPs may soon represent a substantial fraction of industrially produced nanoparticles, necessitating urgent assessment of their potential toxicity [28].

Metallic NP pose ecological risks due to their potential for high environmental concentrations, environmental persistence, and bioaccumulative capacity, largely attributed to their chemical stability and resulting low water solubility [29]. Once introduced into the aquatic environment, Ni-NPs may undergo partial or complete dissolution in the water column, releasing nickel ions, which may remain suspended or settle, either directly or following aggregation. As a result, both suspended Ni-NPs and the released nickel can be ingested by organisms, adhere to external membranes, or be internalized through membrane transport. Upon sedimentation, Ni-NPs can also be ingested or internalized by benthic organisms [30,31]. The impact of these nanoparticles is evident in numerous studies highlighting alterations in cellular and tissue structure and function, as well as in the activity of specific key enzymes [32,33,34]. Ni-NPs exposure may result in an imbalance between reactive oxygen species (ROS) generation and the organism’s antioxidant defense mechanisms, leading to either excessive ROS production or a significant decline in the efficiency of antioxidant defenses [35]. This redox imbalance caused by exposure to environmental stressors may result in oxidative stress and tissue damage, which leads to the accumulation of dysfunctional proteins, lipid peroxidation products, and DNA damage [36,37,38]. Assessment of oxidative stress and damage can be performed through the use of biomarkers, which reveal biological responses at a lower level of biological organization (biochemical level), providing insights into how a substance may affect a particular organism, and offer a rapid and easily quantifiable method to evaluate antioxidant capacity [39]. Oxidative stress represents a key ecotoxicological mechanism through which environmental pollutants exert harmful effects on biological systems. Consequently, various analytical methods have been developed—such as the assessment of superoxide dismutase (SOD), catalase (CAT), glutathione peroxidase (GPx), and glutathione-S-transferases (GSTs) activities, and lipid peroxidation levels [40]. In addition, the detoxification metabolism of nanoparticles often encompasses changes in the glutathione conjugation pathway, and significant increases in the activity of GSTs have been already reported to occur in fish exposed to nickel nanoparticles [41].

The use of organisms from different trophic levels in ecotoxicological and biomonitoring studies offers a broader understanding of contaminant impacts across aquatic food webs [42]. Benthic invertebrates such as *Hediste diversicolor* play a fundamental role in sediment reworking and organic matter cycling, and their ecological relevance, combined with practical advantages like availability and laboratory resilience, make them valuable test organisms [43,44,45]. Filter-feeding bivalves like *Mytilus* spp. are widely recognized bioindicators due to their capacity to accumulate suspended particles, including nanoparticles, and their established role in coastal monitoring programs [46,47,48]. Higher trophic level species, such as fish from the species *Sparus aurata*, a secondary consumer with known bioaccumulation potential, are frequently employed in toxicological research to assess broader ecological and physiological effects [49,50,51].

The main objective of this study was to evaluate the ecotoxicological effects on the three mentioned test organisms, representing different trophic levels (polychaetes, bivalves, and fish), resulting from a chronic exposure (28 days) to ecologically relevant concentrations of Ni-NPs. This was achieved by measuring oxidative stress responses and lipid peroxidation damage (through the determination of CAT and GSTs enzyme activities, and lipid peroxidation levels). The integration of these biochemical responses across different trophic levels will provide a more comprehensive understanding of the ecological impact of Ni-NPs on aquatic ecosystems and their biota.

## 2. Materials and Methods

### 2.1. Reagents and Test Solutions

In this study, commercially available nickel nanoparticles (Ni-NPs) with an average size of ≤100 nm and a purity grade of 99.95% (Sigma-Aldrich, St-Louis, MO, USA. CAS: 7440-02-0) were used. These Ni-NPs were characterized by previous study [52], who reported that (i) the particles were predominantly oval in shape and showed some aggregation, with sizes ranging from 19.30 to 187.27 nm and an average diameter of 76.65 ± 45.38 nm; (ii) transmission electron microscopy (TEM) confirmed particles sizes consistent with the manufacturer’s specification (<100 nm); (iii) in aqueous suspension, the average diameter increased to 217.5 nm due to aggregation; and (iv) the average zeta potential was −24.6 mV, indicating moderate stability and dispersion in water.

The following reagents were used to prepare solutions required for the ecotoxicological assays: 1-chloro-2,4-dinitrobenzene (CDNB; CAS: 97-00-7), solubilized in ethanol (C_2_H_6_O; CAS: 64-17-5); reduced glutathione (GSH; C_10_H_17_N_3_O_6_S; CAS: 70-18-8) for assessing glutathione-S-transferases (GSTs) activity; hydrogen peroxide (H_2_O_2_; CAS: 7722-84-1) for catalase (CAT) activity determination; thiobarbituric acid (TBA; CAS: 504-17-6) and trichloroacetic acid (TCA; Sigma-Aldrich, St. Louis, MO, USA: CAS: 76-03-9) for the lipid peroxidation (TBARS) measurements; and bovine serum albumin (BSA; Sigma-Aldrich, St. Louis, MO, USA. CAS: 9048-46-8) along with Bradford reagent (Bio-Rad UK, Hercules, CA, USA. CA: 94547) for total protein quantification.

A stock suspension of Ni-NPs (100 mg/L) was prepared by dispersing the particles in ultrapure water using a bath sonicator (UP200Ht—Hielscher, Teltow, Germany) for 30 min. The working concentrations (0.05 mg/L, 0.5 mg/L, and 5 mg/L) were obtained by diluting the stock suspension in artificial seawater. Just before use, the suspensions were sonicated again for 30 min to ensure proper dispersion.

### 2.2. Test Organisms and Acclimation

The toxicity of Ni-NPs was assessed through ecotoxicological assays using three species from distinct taxonomic groups and trophic levels to account for potential differences in sensitivity to the nanoparticles: (i) *Hediste diversicolor* (Annelida; Decomposer); (ii) *Mytilus* spp. (Mollusca; Primary Consumer); and (iii) *Sparus aurata* (Chordata; Secondary Consumer).

#### 2.2.1. Polychaetes (*Hediste diversicolor*)

Individuals were manually collected using a metal fork during the low tide from the Douro River, within the Douro Estuary Local Natural Reserve (41.13722 N, 8.66220 W) in northern Portugal, on 5 December 2021. This site is classified as non-polluted (Class I) based on coastal sediment quality guidelines established by the Norwegian Pollution Control Authority [53], which considered background levels of key polycyclic aromatic hydrocarbons and heavy metals [48]. Following collection, polychaetes were transported to the laboratory in plastic buckets containing local brackish water and algae. Upon arrival, organisms were subjected to a two-week quarantine period. During this acclimatation stage, individuals were maintained in 5 L plastic containers containing distilled-washed and air-dried gravel sediment and artificial seawater (Tropical Marine Centre: Premium Reef Salt) with a salinity of 20 ± 2, under constant aeration, at a temperature of 17 ± 1 °C, and a 12 h light/12 h dark photoperiod. Polychaetes were stocked at a density of 200 individuals/m^2^ and seawater was renewed every 48 h. During this period, their body integrity, feeding behavior, and normal movement were monitored. Organisms were fed ad libitum three times a week with commercial fish food (Aquasoja, Sorgal SA, Ovar, Portugal Lisbon, Portugal). At the end of the acclimation period, only apparently healthy individuals with a body mass of 0.500 ± 0.100 g were selected for experimental use.

#### 2.2.2. Mussels (*Mytilus* spp.)

Due to the high levels of hybridization and introgression between *Mytilus edulis* and *M. galloprovincialis* along the Atlantic coast of Europe, the term *Mytilus* spp. is used in this study to avoid potential misidentification or taxonomic ambiguity [48]. Specimens were manually collected during low tide on 25th March 2022 at Agudela Beach, Matosinhos (41.139337° N, 8.655909° W), in northern Portugal. Following collection, mussels were transported to the laboratory in plastic buckets containing aerated seawater and subsequently placed under quarantine for a two-week period. During quarantine, individuals were maintained in 5 L plastic containers under controlled laboratory conditions. Artificial seawater was prepared using Tropical Marin Pro-Reef^®^ marine salt. Experimental parameters included a salinity of 35 ± 2, a temperature of 16 ± 2 °C, a pH of 6.9 ± 0.2, continuous aeration, and a 12 h light/12 h dark photoperiod. Mussels were stocked at a density of 32 individuals per tank. Throughout the acclimation period, mussels were fed twice weekly with a commercial microalgae formulation (Phytobloom Shellbreed, Necton^®^, Faro, Portugal) at a dosage of 0.2 g dry weight per individual. Seawater was renewed every 48 h, and organism viability was monitored. Any deceased individuals were promptly removed.

#### 2.2.3. Fish (*Sparus aurata*)

Specimens of *Sparus aurata* (total length: 6–7 cm) were obtained from the Instituto Português do Mar e da Atmosfera (Olhão, southern Portugal) and transported to the Aquatic Organisms Facility in refrigerated plastic containers with continuous aeration. Upon arrival, fish were transferred to 50 L plastic containers containing aerated, oxygen-saturated seawater and maintained under controlled laboratory conditions for a two-week acclimation period (temperature: 19.0 ± 0.5 °C; pH: 6.9 ± 0.2; dissolved oxygen: 8.7 ± 0.4 mg/L; salinity: 34.5 ± 0.4; ammonia: 0.39 ± 0.07 mg/L; nitrites: 0.40 ± 0.03 mg/L; photoperiod: 12 h light:12 h dark). During acclimation, fish were fed to satiety with a commercial diet (Aquasoja, Sorgal SA, Ovar, Portugal) every two days. No mortality or signs of disease were recorded, and all individuals were deemed healthy and suitable for subsequent experimentation.

### 2.3. Experimental Design and Exposure Conditions

Three independent experiments, each corresponding to a distinct trophic level (polychaetes, mussels, and fish), were conducted under controlled laboratory conditions consistent with those used during the acclimation period. Test organisms were exposed for 28 days to four treatments: a negative control (0.00 mg/L) and three increasing concentrations of Ni-NPs: 0.05 mg/L, 0.50 mg/L, and 5.00 mg/L. These concentrations were selected based on their environmental relevance and in accordance with those used in previous studies [31,54,55]. Chronic exposures were performed following adaptations of the procedures described by guidelines ASTM E1562-00 [56] and ASTM E2455-22 [57] for polychaetes and mussels, respectively, while exposure of fish followed the recommendations inscribed in OECD 215 [58]. Sixty individuals of each test organism were distributed among twelve plastic containers, with three replicates per treatment (i.e., three containers per treatment, each containing five individuals). The containers were randomly arranged within the exposure room. The exposure medium was renewed by 80% every 48 h to maintain water quality and chemical concentration. Water quality was monitored every 48 h throughout the exposure period to ensure test validity. Physicochemical parameters, including pH, temperature, salinity, and dissolved oxygen, were measured using a multiparameter probe (YSI, Yellow Springs, OH, USA: 556 MPS). Water samples were collected for the quantification of ammonia (NH_3_) and nitrites (NO_2_^−^) using a photometer (YSI, Yellow Springs, OH, USA: 9300) and reagent tablets (Palintest, Gateshead, UK: NH_3_ and NO_2_). Values were within the ranges reported during the acclimation period. The use of living organisms in this study was approved by the Animal Welfare and Ethics Board (ORBEA) of CIIMAR. The experimental procedures complied with Portuguese legislation on animal welfare (Decree-Law No. 113/2013). Additionally, one of the supervising researchers holds FELASA Category C certification, granted by the Portuguese Directorate-General for Food and Veterinary Affairs (DGAV), authorizing the use of animals for scientific purposes.

### 2.4. Euthanasia and Collection of Biological Samples

At the end of the 28-day exposure period, test organisms were euthanized for subsequent collection of biological material. Polychaetes were immediately placed in liquid nitrogen and sectioned into approximately equal parts using a scalpel. Mussels were placed on ice, their gills and digestive glands were carefully dissected, and immersed in liquid nitrogen. Fish were euthanized using 2-phenoxyethanol, a method compliant with the AVMA Guidelines for the Euthanasia of Animals and in accordance with Portuguese regulations on animal welfare in experimental procedures (Decree-Law No. 113/2013). Following euthanasia, the fish gills and liver were collected and immediately immersed in liquid nitrogen. All samples were then labeled and stored at −80 °C until further analysis.

### 2.5. Biochemical Determinations

Biological samples previously collected were homogenized in Eppendorf microtubes containing 2 mL of homogenization buffer (50 mM, pH 7.0, with 0.1% Triton X-100: Dow Chemical, Midland, MI, USA), immersed in ice, using a mechanical homogenizer (IKA Ultra-Turrax DI 18 Basic Yellow: Staufen, Germany). Thereafter, the samples were centrifuged at 15,000× *g* for 10 min at 4 °C in a refrigerated centrifuge (Eppendorf 5810R, Hamburg, Germany). The resulting supernatant was collected and stored in Eppendorf microtubes at −80 °C for further analysis, including enzymatic assays [catalase (CAT), glutathione S-transferases (GSTs)] and lipidic peroxidation assessment [thiobarbituric acid reactive substances (TBARS)]. Spectrophotometric measurements (GSTs, CAT, TBARS) were conducted using a Thermo Scientific microplate reader (Waltham, MA, USA), model Multiskan GO, version 1.00.40, with SkanIt Software 3.2. Biomarker quantifications were carried out in triplicate. Dilution factors were taken into consideration.

#### 2.5.1. Determination of Glutathione S-Transferases (GSTs) Activity

GSTs activity in the selected tissues was measured spectrophotometrically according to a valid protocol [59]. The enzymatic conjugation of reduced glutathione (GSH) with the substrate 1-chloro-2,4-dinitrobenzene (CDNB) leads to the formation of a thioether, which can be quantified by the increase in absorbance at 340 nm (ε = 9.6 mM^−1^·cm^−1^). Absorbance readings were taken every 10 s over a period of 5 min. GST activity was expressed as mmol/min/mg protein.

#### 2.5.2. Determination of Catalase (CAT) Activity

CAT activity in the selected tissues was determined following an existent enzymology method [60]. The decomposition of hydrogen peroxide (H_2_O_2_) into water (H_2_O) and oxygen (O_2_) was monitored spectrophotometrically at a wavelength of 240 nm. Catalase activity was assessed by measuring the decrease in absorbance at 240 nm over a 5 min period. The enzymatic activity was expressed as mmol of H_2_O_2_ decomposed per minute per milligram of protein (mmol/min/mg protein).

#### 2.5.3. Quantification of Lipid Peroxidation

Lipid peroxidation was assessed by quantifying the levels of Thiobarbituric Acid Reactive Substances (TBARS) using a spectrophotometric protocol [61]. This method is based on the reaction of malondialdehyde (MDA)—a byproduct of lipid membrane degradation caused by reactive oxygen species (ROS)—with thiobarbituric acid (TBA), forming a colored complex measurable spectrophotometrically at 535 nm (ε = 1.56 × 10^6^ M^−1^·cm^−1^). TBARS concentration was expressed as nanomoles of MDA equivalents per milligram of protein (nmol/mg protein).

#### 2.5.4. Quantification of Total Soluble Protein

Total protein content in all tissue samples was determined based on a dye binding simple method [62]. The binding of Bradford reagent to proteins forms a stable-colored complex, the absorbance of which was measured spectrophotometrically at 595 nm. Protein standards (0.00, 0.25, 0.50, 0.75, 1.00 mg/mL) were prepared using a bovine serum albumin (BSA) stock solution at a concentration of 1 mg/mL.

### 2.6. Statistical Analysis

The data obtained were initially tested for normality using the Shapiro–Wilk test and for homogeneity of variances using Levene’s test. A one-way analysis of variance (ANOVA) was employed to assess biochemical differences among treatments (Factor: Concentration). When significant differences were detected (*p* < 0.05), Dunnett’s post hoc test was applied to identify significant differences between exposed groups and the control group. The significance level was set at 0.05. All statistical analyses were conducted using SigmaPlot version 15.0 (Serial No. 775580169). Results are presented in bar charts as mean ± standard error.

## 3. Results

### 3.1. Glutathione S-Transferases (GSTs)

**Figure 1:**
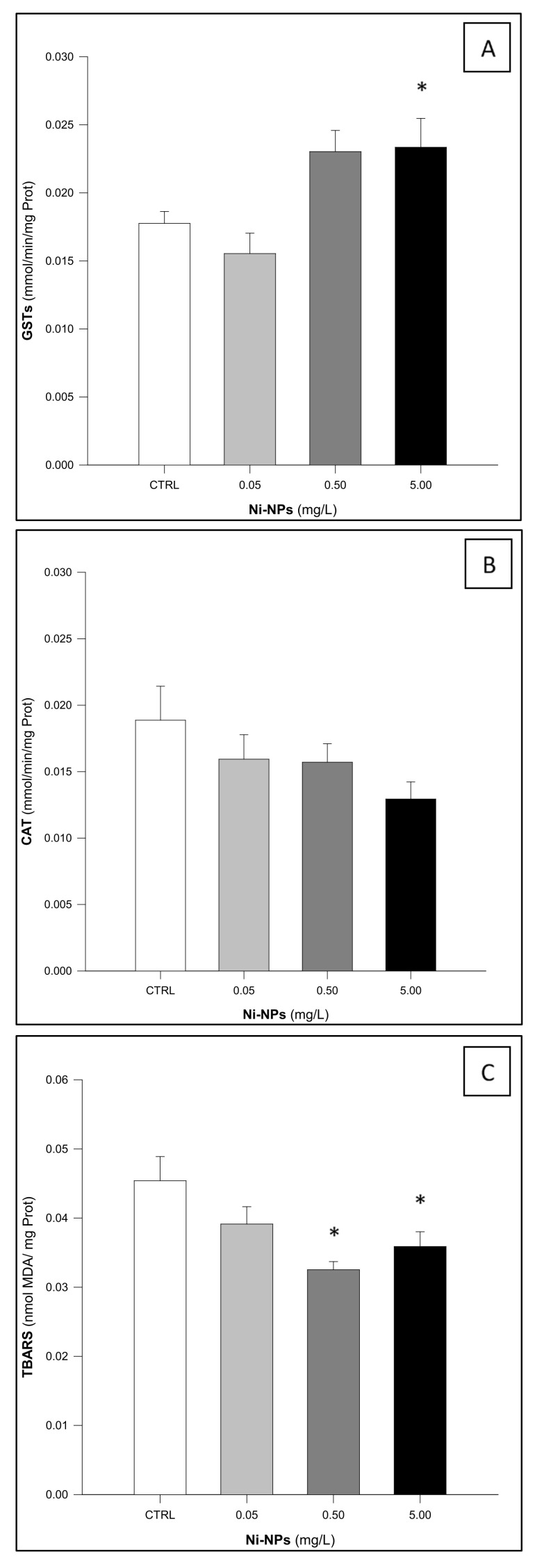
Results of the Glutathione-S-Transferase (GSTs, (**A**)), Catalase (CAT, (**B**)), and Thiobarbituric Acid Reactive Substances (TBARS, (**C**)) in *Hediste diversicolor* following exposure to Ni-NPs (n = 15 animals per treatment). * Indicates statistically significant differences compared to the control group (One-Way ANOVA, followed by a Dunnett’s test, *p* < 0.05).

GSTs activity in *H. diversicolor* samples (Figure 1A) showed statistically significant differences among treatments (One-Way ANOVA: F_3,56_ = 6.055, *p* < 0.05). Notably, individuals exposed to the highest concentration of Ni-NPs (5.00 mg/L) exhibited a significant increase in the enzymatic activity when compared to the control group (Dunnett’s test: *p* < 0.05).

**Figure 2:**
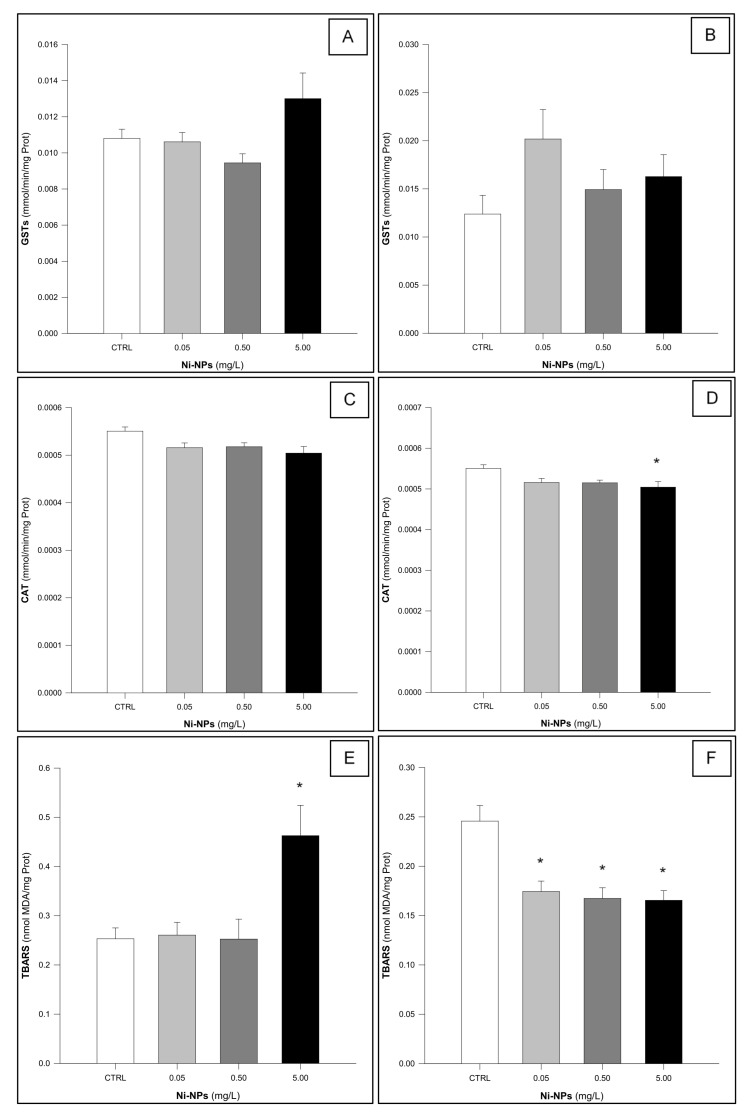
Results of the Glutathione-S-Transferase (GSTs: (**A**,**B**)—gills and digestive gland, respectively), Catalase (CAT: (**C**,**D**)—gills and digestive gland, respectively), and Thiobarbituric Acid Reactive Substances (TBARS: (**E**,**F**)—gills and digestive gland, respectively) in *Mytilus* spp. following exposure to Ni-NPs (n = 15 animals per treatment). * Indicates statistically significant differences compared to the control group (One-Way ANOVA, followed by a Dunnett’s test, *p* < 0.05).

GSTs activity in the gills of *Mytilus* spp. following chronic exposure (Figure 2A) showed a significant difference among treatments (One-Way ANOVA: F_3,56_ = 3.159, *p* = 0.032). However, none of the exposed groups differed significantly from the control group (Dunnett’s test: *p* > 0.05). A similar result was observed in the digestive gland (Figure 2B), where no statistically significant differences were found among treatments (One-Way ANOVA: F_3,56_ = 1.873, *p* = 0.145).

**Figure 3:**
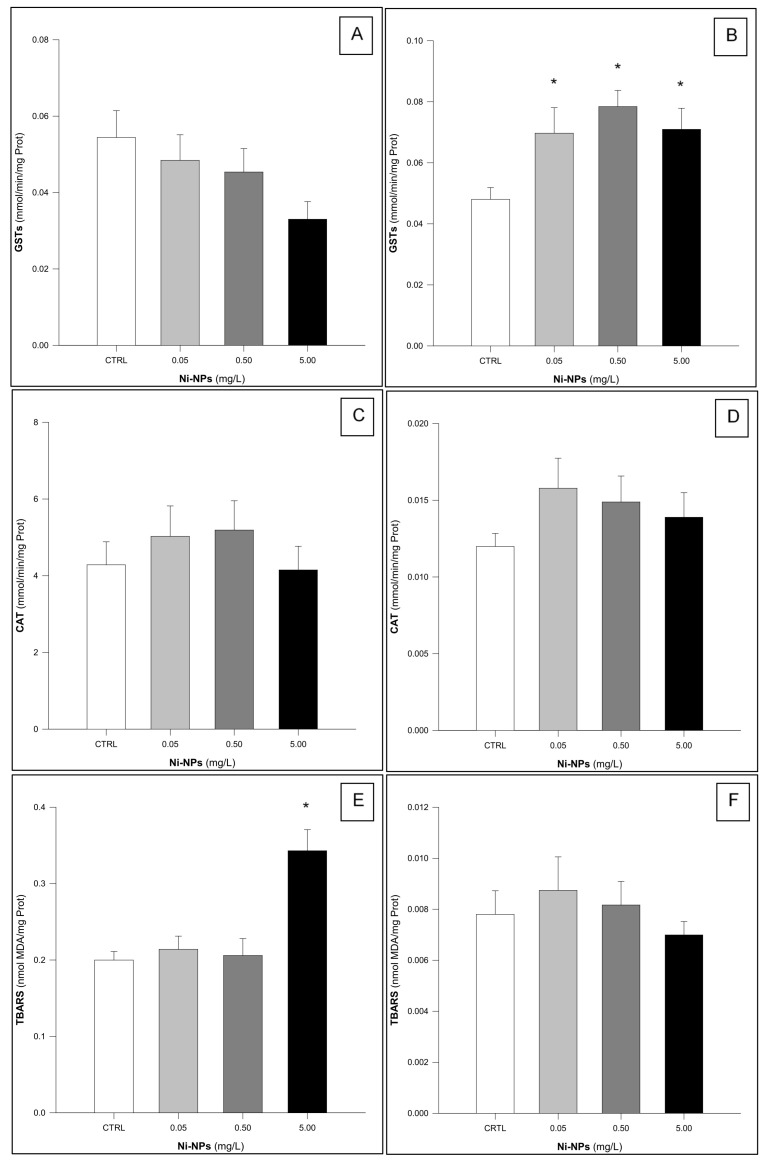
Results of the Glutathione-S-Transferase (GST: (**A**,**B**)—gills and liver, respectively), Catalase (CAT: (**C**,**D**)—gills and liver, respectively), and Thiobarbituric Acid Reactive Substances (TBARS: (**E**,**F**)—gills and liver, respectively) in *Sparus aurata* following exposure to Ni-NPs (n = 15 animals per treatment). * Indicates statistically significant differences compared to the control group (One-Way ANOVA, followed by a Dunnett’s test, *p* < 0.05).

GSTs activity in the gills of *S. aurata*, organisms exposed to Ni-NPs did not show statistically significant differences among treatments (Figure 3A) (One-Way ANOVA: F_3,56_ = 2.129, *p* = 0.107). This was not the case in liver samples (Figure 3B), where statistically significant increases were observed in GSTs activity measured in organisms exposed to Ni-NPs (One-Way ANOVA: F_3,56_ = 4.249, *p* < 0.05). Furthermore, individuals in the exposed groups exhibited increased GSTs activity compared to the control group (Dunnett’s test: *p* < 0.05).

### 3.2. Catalase (CAT)

CAT activity in *H. diversicolor* (Figure 1B) did not show significant differences among treatments (One-Way ANOVA: F_3,56_ = 1.739, *p* = 0.170), despite an apparent decrease in catalase activity with increasing concentrations of Ni-NPs, but not statistically significant.

CAT activity in the gills of *Mytilus* spp. showed no significant differences among treatments (One- Way ANOVA: F_3,56_ = 0.804, *p* = 0.497) (Figure 2C). In contrast, CAT activity in the digestive glands of Mytilus spp. showed a statistically significant difference among treatments (One-Way ANOVA: F_3,56_ = 3.602, *p* < 0.05). Specifically, exposed individuals exposed to the highest concentration of Ni-NPs exhibited reduced catalase activity compared to the control group (Dunnett’s test: *p* < 0.05) (Figure 2D).

CAT activity in the gill tissue of *S. aurata* did not exhibit significant differences among treatments (One-Way ANOVA: F_3,56_ = 0.556, *p* = 0.646) (Figure 3C). A similar result was also observed for CAT activity in the hepatic tissue (One-Way ANOVA: F_3,56_ = 1.064, *p* = 0.372) (Figure 3D).

### 3.3. Thiobarbituric Acid Reactive Substances (TBARS)

*H. diversicolor* individuals showed significant differences among treatments regarding TBARS (One-Way ANOVA: F_3,56_ = 6.984, *p* < 0.05), since the individuals exposed to the two highest concentrations of Ni-NPs exhibited significantly lower levels of lipid peroxidation compared to the control group (Dunnett’s test: *p* < 0.05) (Figure 1C).

TBARS levels in the gills of *Mytilus* spp. (Figure 2E) showed a statistically significant increase (One-Way ANOVA: F_3,56_ = 6.500, *p* < 0.05), but only for individuals exposed to the highest concentration of Ni-NPs (5.00 mg/L) (Dunnett’s test: *p* < 0.05). TBARS levels in the digestive gland also showed significant differences among treatments (One-Way ANOVA: F_3,56_ = 10.407, *p* < 0.05), with statistically significant lower levels observed between all exposed groups and the control group (Dunnett’s test: *p* < 0.05) (Figure 2F).

Lipid peroxidation levels in gill samples of *S. aurata* (Figure 3E) differed significantly among treatments (One-Way ANOVA: F_3,56_ = 11.224, *p* < 0.05). A significant increase in TBARS levels was observed only in individuals exposed to the highest concentration of Ni-NPs compared to the control group (Dunnett’s test: *p* < 0.05). In contrast, TBARS levels in the hepatic tissue of *S. aurata* did not show significant differences among treatments (One-Way ANOVA: F_3,56_ = 0.581, *p* = 0.830) (Figure 3F).

## 4. Discussion

This study evaluated the potential of Ni-NPs to interfere with oxidative stress and metabolic biomarkers such as glutathione-S-transferases (GSTs) and catalase (CAT), which are enzymes involved in phase II metabolism and antioxidant defenses, respectively. Additionally, levels of thiobarbituric acid reactive substances (TBARS)—a key biomarker of lipid peroxidation in polyunsaturated fatty acid-rich tissues—were measured. The results demonstrated that Ni-NPs exposure impacted oxidative stress, metabolism and lipid peroxidation biomarkers (GSTs, CAT, and TBARS) in a species- and tissue-specific manner.

Ni-NPs are known for their capacity to cause oxidative related toxic effects, in distinct animal models, as recently reviewed [63]. In several fish species, this pattern of toxic response has also been observed, as described in *Oreochromis mossambicus* [32], in *Danio rerio* [35], in *Labeo rohita* [64], and in *Carassius auratus* [65], among others. Similar outcomes have also been reported to occur in fish cell lines, namely in *Lepomis macrochirus* (BF-2) cells [41]. Furthermore, other aquatic species have also been shown to be susceptible to Ni-NPs, with oxidative stress identified as a key mechanism of toxicity. Notable examples include the freshwater macrophyte *Lemna gibba* [66], the microalgae *Pseudokirchneriella subcapitata* [67], the marine calanoid copepod *Centropages ponticus* [68], and the marine mussel *Mytilus galloprovincialis* [55]. Therefore, it is possible to suggest that oxidative stress, or the activation of the antioxidant defense mechanisms, is a common response among many distinct taxa.

However, the here obtained data show a somewhat different pattern of response, which was highly species specific. Notably, considering GSTs activity, no significant changes were detected in the gills and digestive gland of *Mytilus* spp., suggesting the absence of phase II conjugation pathway involving glutathione, or the increase in the production of ROS. On the contrary, GSTs activity increased significantly in *H. diversicolor*, mirroring results from polluted environments and exposures to various metal nanoparticles [69], including Ni-NPs. Similarly, significant GSTs activation was observed in the liver of *S. aurata*, the main detoxification fish organ, likely indicating an active role in Ni-NPs biotransformation, suggesting that GSH plays a central role as chelating agent, antioxidant and signaling component [70,71]. This increase may explain the hereby observed CAT inactivation (as discussed below) or its activation beyond a threshold of oxidative stress [72]. In contrast, GST activity in the gills of *S. aurata* showed a non-significant trend toward inhibition, possibly due to oxidative stress-induced GSH depletion, direct interaction with the enzyme’s structure, or changes in gene expression [72,73,74] following Ni-NPs chronic exposure. In general, the observed changes, except for *Mytilus* spp., corroborate the body of data from the literature showing that Ni-NPs are causative of deleterious changes that are directly related to the increase in the production of ROS. Mussels in general, namely those from the *Mytilus* genus, are well known for their strategy to avoid the interaction with waterborne chemical contaminants, which involves the closing of their valves, and thereby preventing the absorption of environmental toxicants. This physiological response, which is primarily triggered in the presence of suspended microalgae [75], may also be triggered in the presence of toxicants, such as metals [76], pesticides [77], crude oil [78], and oils dispersants [79]. Being an unspecific response to adverse conditions, it is possible to suggest that exposed mussels deployed a similar mechanism, which prevented or minimized the uptake of Ni-NPs from the external media, and the absence of biological responses in terms of GSTs activity.

The activity of CAT in aquatic organisms seems to be upregulated following exposure to Ni-NPs, as recently demonstrated to occur in the mussel species *Mytilus galloprovincialis* [55] and in the fish species *Heteropneustes fossilis* [34]. However, this trend seems to depend on the studied species, since inhibition of CAT activity has also been documented in freshwater species such as *Carassius auratus* and *Labeo rohita* upon exposure to CeO_2_-NPs and NiO-NPs, respectively [11,64]. The hereby results show a pattern consistent with this later trend, since this enzyme was significantly impaired in the digestive gland of Mytilus spp. for the individuals exposed to the higher Ni-NPs concentration. In *H. diversicolor*, CAT activity showed a dose-dependent but non-significant decrease. This inhibition, consistent with findings by previous works [55,80] suggests possible direct structural damage to the enzyme, antioxidant system exhaustion, or impaired conversion of superoxide anions due to SOD dysfunction [81]. Conversely, some studies have observed increased CAT activity in *Oncorhynchus mykiss* following exposure to CeO_2_-NPs [17] underscoring interspecies variability, and the possibility that different nanoparticles can yield distinct effects in terms of the antioxidant defense system. However, no significant CAT activity changes were observed in both tissues of *S. aurata*, possibly due to the activation of alternative compensatory antioxidant mechanisms, such as SOD, GPx, or GSTs [82,83], to cope with the excess of ROS resulting from Ni-NPs.

TBARS levels, used to assess lipid peroxidation, increased significantly in the gills of *Mytilus* spp. of the individuals exposed to the higher concentration of Ni-NPs, potentially indicating that contaminant concentrations exceeded detoxification thresholds, leading to pro-oxidant effects [84]. Conversely, TBARS levels decreased in the digestive gland of *Mytilus* spp. and in *H. diversicolor* of the exposed individuals, likely due to compensatory GPx and GSTs activity [85,86]. In addition, exposure to Ni-NPs resulted in a dose-dependent increase in lipid peroxidation in gills of *S. aurata* exposed to the higher concentration of the tested toxicants, while no changes were reported in liver tissue of the same organisms. It is thus possible to suggest that, despite the activation of an antioxidant response, as previously discussed, this response was not effective enough to deal with the excessive production of ROS resulting from Ni-NPs metabolism, which culminated in tissue oxidative damage, especially in *Mytilus* spp., and *S. aurata*. This effect is in line with previously published data, for distinct taxa, including human cell lines [87,88,89] rodents [63,90,91], and in fish [34,35], but also in aquatic invertebrates, such as ascidians [92], and microalgae [93]. Therefore, it is possible to suggest that exposure to Ni-NPs, even in ecologically relevant conditions, are causative of oxidative damage, which is not effectively prevented by the triggering of antioxidant defensive mechanisms, such as those previously discussed. The establishment of oxidative damage, due to the overall inefficacy of the antioxidant defense, may lead to the impairment of the natural fluidity and flexibility of biological membranes [94,95], compromising key cellular functions, favoring aging, resulting in the onset of varied pathological processes [96], and ultimately leading to cell death. Considering the response of organisms of different trophic levels, it is possible to suggest that Ni-NPs may exert deleterious effects that are transversal to the entire ecosystem, thereby adversely affecting ecological functions.

## 5. Conclusions

The increasing use of metallic nanoparticles, including Ni-NPs, raises concerns regarding their impact on aquatic ecosystems. This study demonstrates species- and tissue-specific alterations in antioxidant defense mechanisms and lipid peroxidation levels following Ni-NPs exposure. These effects may compromise key physiological features of environmentally exposed organisms, from distinct trophic levels, which perform essential ecological functions, leading to a potential disruption of trophic dynamics. For example, reduced physiological efficiency in *Mytilus* spp. may impair filtration capacity, affecting water clarity and phytoplankton composition. Similarly, disruptions in *H. diversicolor* may alter nutrient cycling and sediment oxygenation due to its bioturbation role. As a top predator, *S. aurata* plays a regulatory role in estuarine and marine food webs, and its impairment could impact biodiversity and ecosystem resilience. Overall, the findings suggest that Ni-NPs may pose a toxicological risk to marine organisms with ecological consequences, even when exposure occurs to ecologically relevant levels. However, the study focused on a limited set of oxidative stress biomarkers and did not assess bioaccumulation or trophic transfer processes. Future research should explore additional molecular pathways, physiological mechanisms and long-term ecological impacts.

## Data Availability

The original contributions presented in this study are included in the article. Further inquiries can be directed to the corresponding author.
